# Friction and Wear Properties of Ni_3_Si Alloy with Ti Addition at High Temperatures

**DOI:** 10.3390/ma13040982

**Published:** 2020-02-22

**Authors:** Changyin Wu, Muye Niu, Shuai Bao, Yuhang Sun, Xinghua Zhang

**Affiliations:** 1 School of Materials Science and Engineering, Jiangsu University of Science and Technology, Zhenjiang 212003, Chinasyh951214@163.com (Y.S.); 2 National Demonstration Center for Experimental Materials Science and Engineering Education (Jiangsu University of Science and Technology), Zhenjiang 212003, China

**Keywords:** tribological properties, Ni_3_Si alloy, wear mechanisms, high temperatures

## Abstract

The tribological properties of Ni_3_Si alloy were studied at high temperatures. The effect of the addition of Ti was also analyzed. The surface composition was analyzed by Raman spectroscopy. The results showed that the friction coefficient decreased with the increasing temperature, and the wear rate changed slightly from 25 to 400 °C. However, the wear resistance of the alloys decreased sharply at 600 °C, and this was due to the decrease of the high-temperature strength and the severe oxidation of the alloys. Although the oxidation resistance of Ni_3_Si alloy decreased with Ti addition, the tribological property was improved by the addition of Ti. The Ni_3_Si alloy with 5% Ti addition had the best wear resistance at high temperatures as compared to pure Ni_3_Si alloy and with 10% Ti addition, and the wear rates of the alloys were in the order of magnitude of 10^−5^ mm^3^/Nm. With the increase of temperature, the wear mechanism of pure Ni_3_Si alloy transformed from abrasive wear to oxidation wear. As the Ti content increased, the wear mechanisms of the alloys changed from abrasive wear to fatigue wear at low temperature, and oxidation wear and fatigue wear at high temperature.

## 1. Introduction

There is an increasing demand for a wide range of structural materials for high strength, excellent oxidation resistance, low density, good wear, and corrosion resistance. Unfortunately, few kinds of alloys can satisfy all these conditions simultaneously. However, intermetallic compounds have specific mechanical and chemical properties, which can meet these performance requirements. For example, Ni_3_Si alloy possesses an anomalous hardness–temperature relationship (an increase in strength with increasing temperature) and excellent oxidation resistance over a wide range of temperatures [1,2,3,4,5]. In addition, it also exhibits excellent corrosion resistance in sodium chloride and various acidic solutions [6,7,8,9]. Therefore, the alloy is the potential to be used in structural or chemical components, especially in high-temperature fields. Further, it is necessary to study the tribological property of the alloy under harsh environmental conditions.

Bi et al. fabricated the Ni_3_Si alloy with Cr addition by combustion synthesis and the tribological properties of the alloy were investigated under high temperature, sulfuric acid solution, dry and water conditions [10,11,12,13]. The results showed that the wear rates of the alloy were in the magnitude of 10^−5^ mm^3^/Nm at the temperature below 800 °C; similarly, the wear rates were in the order of 10^−5^ mm^3^/m under low and moderate loads both in sulfuric acid solution and water. This indicated that Ni_3_Si alloy possessed excellent wear resistance under these conditions. Niu et al. investigated the tribological properties of Ni_3_Si alloy with Ti addition under vacuum and seawater conditions [14,15]. These results demonstrated that the alloy possessed lower friction coefficient and wear rate in low vacuum condition as compared to high vacuum and air conditions; the tribological properties of Ni_3_Si alloys with different Ti addition are better than that of Ti6Al4V alloy under seawater condition; in addition, the counterpart materials had a significant effect on the tribological behaviors of the alloys, and the order of friction coefficients was Ni_3_Si-Si_3_N_4_ > Ni_3_Si-316SS > Ni_3_Si-Al_2_O_3_. 

Gui et al. studied the tribological properties of NiMo/Mo_2_Ni_3_Si intermetallic composites under dry sliding wear test conditions at room temperature, and found that the wear mass losses and the friction coefficients of the composite were considerably lower than those of the comparison test materials, the austenitic stainless steel 1Cr18Ni9Ti, and hardened 0.45%C steel [16]. Wang et al. observed that Moss-toughened Mo_2_Ni_3_Si alloys exhibited excellent wear resistance and low friction coefficient under dry sliding conditions [17]. Especially under high load, the wear resistance of the alloy was 10 times more than that of hardened 0.45%C steel. Wang et al. also investigated the tribological properties of Ti_2_Ni_3_Si/NiTi intermetallic composite coating and the high-temperature sliding wear resistance of the γ-toughened Cr_13_Ni_5_Si_2_ alloy [18,19]. The Ti_2_Ni_3_Si/NiTi coating had excellent abrasive and adhesive wear resistance under dry sliding conditions, and the wear loss reduced by more than 15 times when compared with the hardened 0.45%C carbon steel and the hardened high-carbon low-alloy tool-steel 1.0%C–1.5%Cr. Wear volume loss of the Cr_13_Ni_5_Si_2_ alloy was dramatically lower than that of the austenitic steel 1Cr18Ni9Ti and was even slightly decreased with the increasing test temperature. 

From the abovementioned work, the study on the tribological properties of Ni_3_Si alloy mainly focused on the effect of alloying elements of titanium and chromium. However, with the addition of Cr, Ni_3_Si alloy was apt to form a brittle phase, and this led to increased strength and decreased toughness [20]. On the contrary, the strength and ductility of Ni_3_Si alloy increased with Ti addition [2], but the high-temperature tribological properties of the alloy were rarely studied. The reason might be that the oxidation resistance of Ni_3_Si alloy declined slightly with the decrease of Si content [21]. It is noted that a high content of oxides is conducive to reduce friction and wear [22,23]. Therefore, the research of high-temperature tribological properties of Ni-Si-Ti alloy will provide a comprehensive understanding of the alloy, which is beneficial to the application of the alloy in high-temperature service condition. In the present work, the wear resistance and friction coefficient of Ni_3_Si alloy with Ti addition were evaluated at high temperatures. The effects of the temperature and the content of Ti were discussed, and the corresponding wear mechanisms were analyzed.

## 2. Materials and Methods 

The Ni_3_Si alloys with different Ti addition were prepared by mechanical alloying and vacuum hot-pressing sintering. The preparation process is described in detail elsewhere [24]. The tribological tests were carried out by a ball-on-disk tribometer with the alloys and bearing steel balls as a counter material. The alloy disk with a dimension of 18 × 18 × 3 mm^3^ was rotated against a commercial Si_3_N_4_ ceramic ball (diameter of 5 mm, hardness of 15 GPa, roughness of 0.02 μm). Before each experiment, the specimens were ground with SiC abrasive paper and polished with diamond abrasive to Ra ≤ 0.06 μm, followed by ultrasonic cleaning in an acetone solution. The applied loads were 10 N, and the sliding speed was 0.10 m/s. The total sliding time was 20 min. The test temperatures were 25, 200, 400, and 600 °C. The wear rate was calculated by the expression: W = SC/NL, where S is the cross-sectional area, C is wear track perimeter, N is the applied load, and L is sliding distance. The cross-sectional profile of the worn surface was measured using a confocal laser scanning microscope (LEXT OLS4000, Tokyo, Japan), and the area was calculated automatically by the equipment. All the tests were carried out at least three times under identical conditions. A scanning electron microscope (SEM, JSM-5600LV, Tokyo, Japan) equipped with energy dispersive spectroscopy (EDS) (Oxford Instruments, Abingdon, UK) was used to study the morphology characteristics and chemical composition of the worn surfaces. The SERS measurement was carried out with a Renishaw InVia Raman microscope (Gloucestershire, UK).

## 3. Results and Discussion

### 3.1. Tribological Behaviors of the Alloys

**Figure 1:**
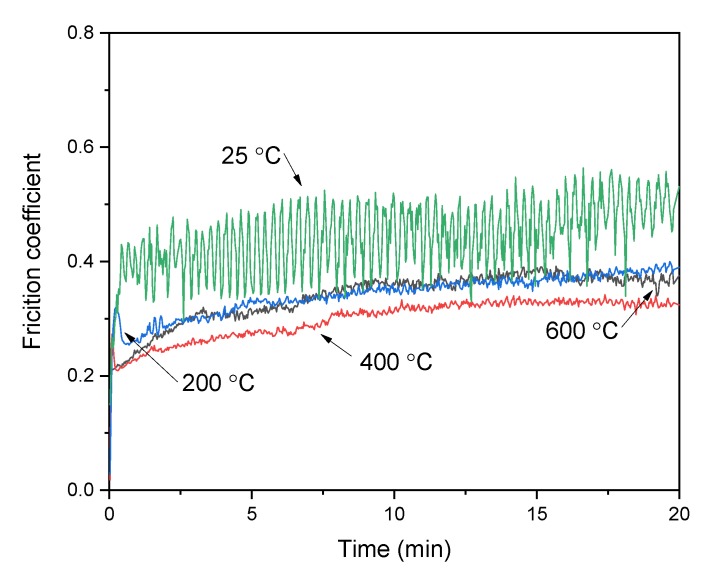
Variation curves of friction coefficient of Ni_3_Si alloy with 10% Ti addition against Si_3_N_4_ at a load of 10 N and sliding speed of 0.10 m/s.

The typical curves of friction coefficients (COFs) as a function of time are shown in Figure 1. It was clear that the COFs became stable after a short running-in period, and the value was higher at room temperature as compared to high temperatures. The fluctuations of the friction coefficient curves were relatively low at high temperatures, the reason for this might be that the oxides played a critical role in reducing friction and maintaining the friction force stable, which formed during the sliding process at high temperature. The friction coefficient of the alloy with 10% Ti addition was lowest at 400 °C.

**Figure 2:**
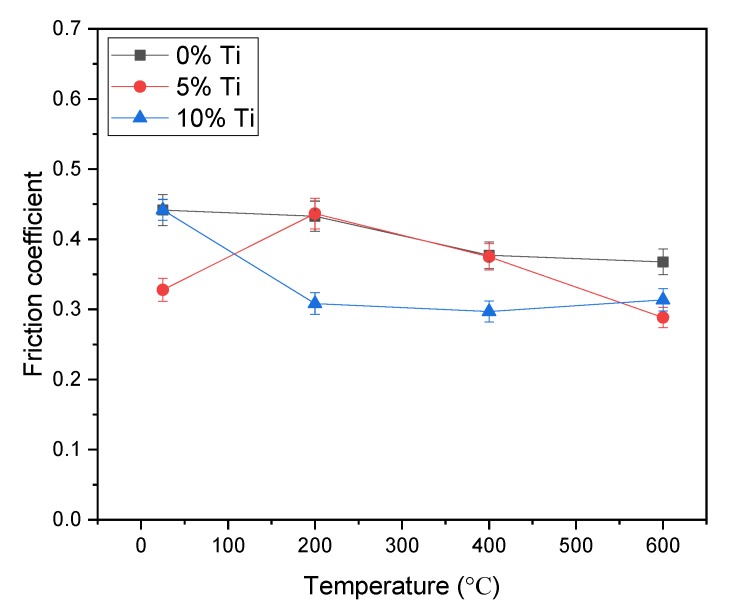
Average friction coefficients of the alloys at different temperatures.

The friction coefficient as a function of temperature for Ni_3_Si alloy is given in Figure 2. As the temperature increased, the friction coefficient of pure Ni_3_Si alloy showed a descending trend. This could be related to the good high-temperature mechanical properties and the oxidation of the alloy at high temperatures. With the addition of 5% Ti, the alloy had a low friction coefficient at room temperature, and a higher friction coefficient at 200 °C. Then, the friction coefficient decreased with the temperature rising. The Ni_3_Si alloy with 10% Ti addition exhibited an obvious decrease in friction coefficient from room temperature to 400 °C, and a slight increase in friction coefficient at 600 °C. The reverse trend between the two alloys was due to the different content of Ti addition.

**Figure 3:**
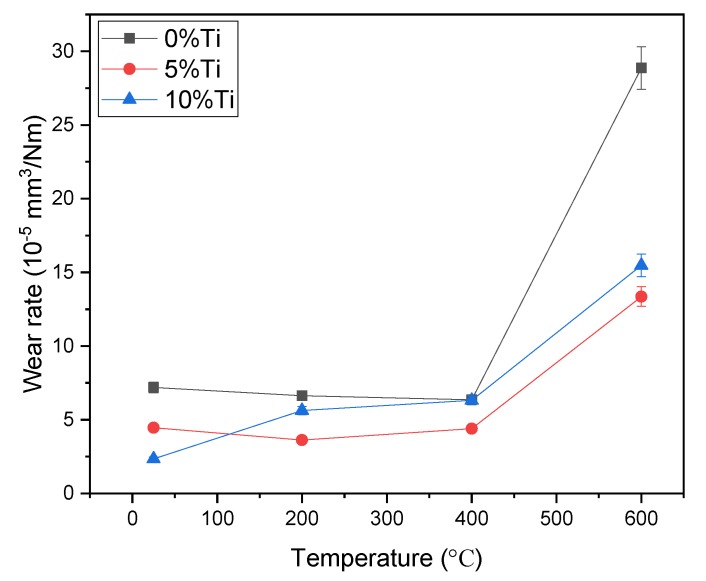
Wear rates of the alloys at different temperatures.

The wear rate as a function of temperature for Ni_3_Si alloy is shown in Figure 3. The wear rate of pure Ni_3_Si alloy decreased slightly in the temperature range from 25 to 400 °C. The alloy with 10% Ti addition had the lowest wear rate at room temperature, and a little lower wear rate at the medium temperature as compared to pure Ni_3_Si alloy. The alloy with the addition of 5% Ti exhibited the lowest wear rate at high temperatures, and showed the optimal wear resistance among the tested materials. When the temperature rose from 400 to 600 °C, the wear rates of the alloys all increased sharply, especially pure Ni_3_Si alloy. It was noted that the wear rates of the alloys were in the magnitude of 10^−5^ mm^3^/Nm.

### 3.2. Wear Mechanisms of the Alloys

**Figure 4:**
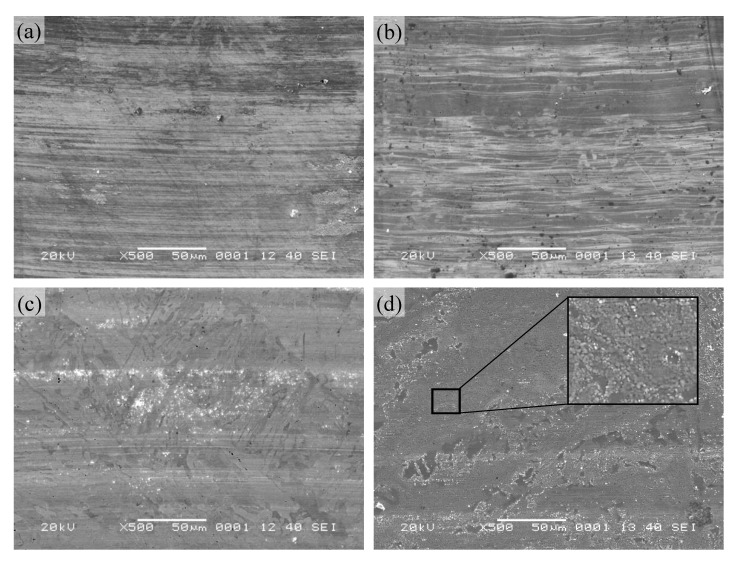
SEM micrographs of worn surface of pure Ni_3_Si alloy at different temperatures: (**a**) 25; (**b**) 200; (**c**) 400; (**d**) 600 °C.

The surface topographies of the wear tracks of pure Ni_3_Si alloy are displayed in Figure 4. It was clear that there were numerous scratches and furrows on the worn surface in the temperature range from 25 to 200 °C (Figure 4a,b). This indicated that the wear mechanisms of pure Ni_3_Si alloy were abrasive wear at these temperatures. When the temperature was up to 400 °C, some shallow grooves, as well as fine wear debris existed on the worn surface (Figure 4c). The main wear mechanism was abrasive wear, and accompanied with oxidation wear at the temperature. At 600 °C, the worn surface was covered with a compact oxide film, and the composition of the oxide was 51.83Ni-14.83Si-33.34O. However, the oxide film was discontinuous with granular structure, and unable to effectively protect the sublayer. Therefore, the alloy suffered severe oxidation wear, and the wear rate of the alloy rose rapidly at this temperature. In addition, for pure Ni_3_Si alloy, the yield strength was highest at 400 °C, and then decreased significantly [25]. On that basis, the wear rate and the friction coefficient of the alloy decreased from 25 to 400 °C. At the temperature of 600 °C, the oxidation of the alloy occurred obviously, and the granular oxide layer formed and covered on the entire surface (Figure 4d). The oxide layer did not form a glaze layer on the worn surface and tended to peel off. Combined with the decreased strength above 400 °C, the friction coefficient of the alloy decreased slightly, and the wear rate had a dramatic increase.

**Figure 5:**
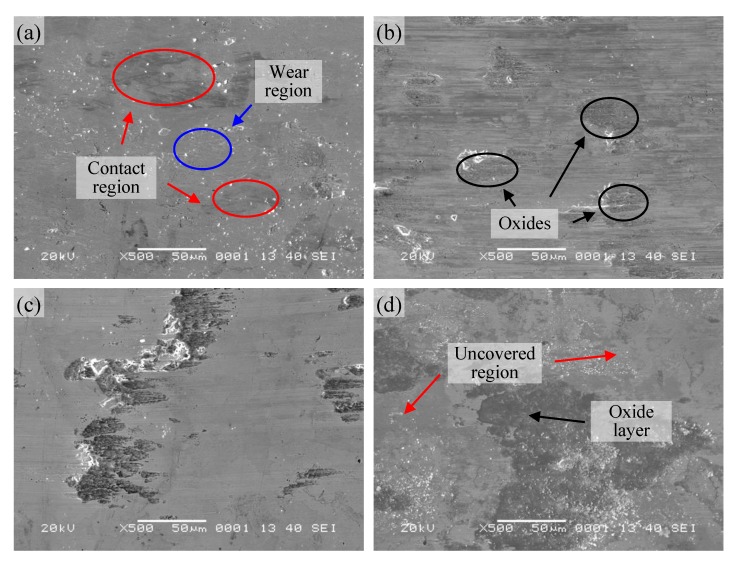
SEM micrographs of worn surface of Ni_3_Si alloy with 5% Ti addition at different temperatures: (**a**) 25; (**b**) 200; (**c**) 400; (**d**) 600 °C.

The morphologies of the wear scars of Ni_3_Si alloy with 5% Ti addition are shown in Figure 5. The worn surface of the alloy was divided into two regions at room temperature: A contact region and a wear region (Figure 5a). The compositions of the contact region and the wear region were 58.4Ni-16.3Si-14.2Ti-10.0O and 68.2Ni-26.2Si-5.6O, respectively. This was because of the harder phase Ni_3_Ti (about 700 HV) formed during the sintering process, which was able to support the applied load effectively [24]. In contrast, the Si-rich region (the wear region, about 500 HV) had lower hardness, and was worn out firstly. As a result, the wear resistance of the alloy was improved as compared to pure Ni_3_Si alloy, and the real area of contact was smaller during the sliding process. The alloy showed a relatively low friction coefficient and wear rate at room temperature, and the wear mechanism was slightly abrasive wear. As the temperature rose to 200 °C, the worn surface was covered with some oxides besides some shallow grooves (as shown in Figure 5b). The composition of the oxide was 30.7Ni-14.8Si-4.2Ti-50.4O, Ti content is relatively high. Along with the oxidation of Ti-rich region, the real area of contact increased, and so the alloy had a high friction coefficient at this temperature. Due to the good high-temperature mechanical property of Ni_3_Si alloy and the compacted oxide wear debris, the wear rate of the alloy was lower at 200 °C. The wear mechanism was abrasive wear and oxidation wear. 

When the temperature reached 400 °C, the worn surface was smooth, and covered with oxides in localized areas (shown in Figure 5c). Like pure Ni_3_Si alloy, the smooth surface was attributed to the highest strength at 400 °C, and the difference is the formation of the oxide layer. This indicated that although the oxidation resistance of the alloy with 5% Ti addition decreases, the wear rate of the alloy was lower. The wear mechanism was oxidation wear at the temperature. At 600 °C, the oxide layer formed (Figure 5d), which composition was 30.4Ni-9.7Si-2.3Ti-57.6O. The uncovered area had been worn out and was re-oxidized during the sliding process. The EDS result showed that the composition of this area is 57.5Ni-19.3Si-1.4Ti-21.8O, which had a low oxidation degree. The significant oxidation and wear resulted in a low friction coefficient and high wear rate simultaneously as compared to that at 400 °C. It was worth pointing out that the morphology of the worn surface of the alloy with 10% Ti addition was quite different from that of pure Ni_3_Si alloy at 600 °C. The continuous dense oxide layer (Figure 4d) showed the better oxidation resistance of pure Ni_3_Si alloy than the alloy with Ti addition. However, the better oxidation resistance was, the less oxides formed. It was well-known that a lot of oxides were compressed and smeared into the surface during the sliding process, and this continued until the sintered surface layer of thickness was sufficient to prevent further disruption of the alloy surface [26]. Although the oxidation resistance decreased with the addition of Ti, the tribological property of the alloy with Ti addition was improved as compared to pure Ni_3_Si alloy. Therefore, the friction coefficient and wear rate of the alloy with 5% Ti addition were lower than those of pure Ni_3_Si alloy at this temperature.

**Figure 6:**
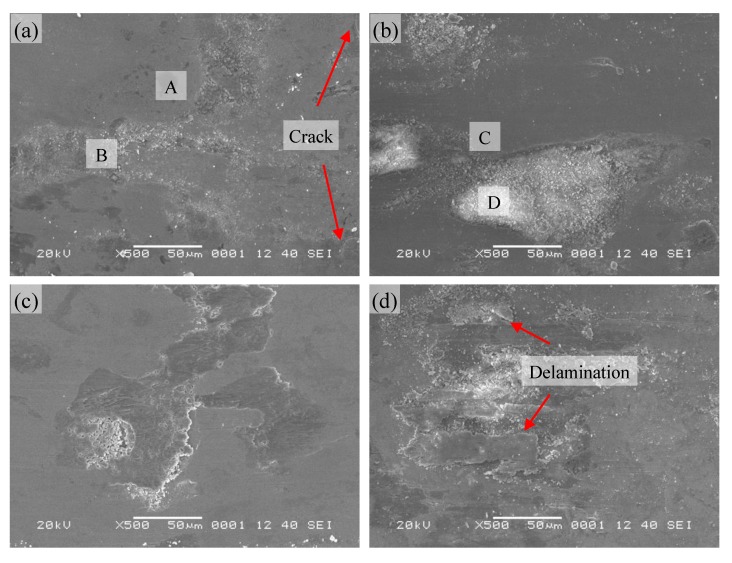
SEM micrographs of worn surfaces of Ni_3_Si alloy with 10% Ti addition at different temperatures: (**a**) 25; (**b**) 200; (**c**) 400; (**d**) 600 °C.

The morphologies of the wear scars of Ni_3_Si alloy with 10% Ti addition are given in Figure 6. The compositions of A and B regions were 61.2Ni-6.3Si-24.4Ti-8.0O and 31.0Ni-7.2Si-2.8Ti-59.0O, respectively (shown in Figure 6a). With the increase of Ti content, the area of the Ti-rich region (A region) increased, and the morphology of the region was relatively smooth without obvious grooves. The smoother surface caused the lower wear rate and the higher real area of contact. Therefore, Ni_3_Si alloy with 10% Ti addition showed a high coefficient of friction and a low wear rate simultaneously at room temperature. It was noted that the worn surface appeared some wear debris and cracks, and the fluctuation of the friction coefficient might be attributed to the interaction of wear debris and localized fracture of the tribolayer [27,28]. The main wear mechanism was oxidation wear, along with fatigue wear at room temperature. The elementary compositions of C and D regions were 62.3Ni-14.1Si-13.4Ti-10.3O and 27.6Ni-7.7Si-3.4Ti-61.4O, respectively (shown in Figure 6b). A large number of fine oxide particles stacked on the worn surface, and played a role in reducing friction. Although the Ti-rich region was smooth, the area of the flake pits was larger, and thus the alloy with 10% Ti addition had a moderate wear rate among the tested alloys at 200 °C. 

As the temperature rose further, the oxide layer formed on the worn surface, and the uncovered area was smooth, which was similar to that of pure Ni_3_Si alloy and with 5% Ti addition at the same temperature. The compact oxide layer and the smooth surface maintained the friction coefficient and wear rate at a low level at 400 °C (Figure 6c). When the temperature reached 600 °C, the oxidation was further accelerated, the oxides were swept out of the wear track before the oxide layer could form on the worn surface (Figure 6d). The presence of oxide particles was helpful to have a low level of friction coefficient. At the same time, the intense oxidation brought severe delamination for the worn surface. Further, the wear rate of the alloy with 10% Ti addition increased remarkably at 600 °C.

### 3.3. Raman Spectrum Analyses of the Alloys

**Figure 7:**
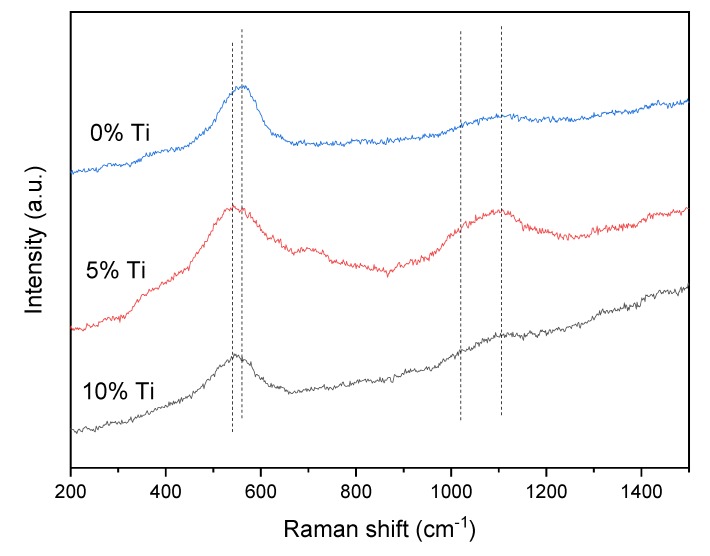
Raman spectra of the alloys with the addition of different Ti contents at 600 °C.

The Raman spectra of the alloys at 600 °C are shown in Figure 7. It was observed that all the Raman spectra of the alloys had a broad intense Raman band in the 700–400 cm^−l^ region. The bands in this region are associated with the presence of bridging oxygens (Si–O–Si) in the structure [29]. This indicated that the worn surface was heavily oxidized at high temperature. However, the compositions of the surface of the alloys were different. For pure Ni_3_Si alloy, the Raman peak was located at 560 cm^−1^, and the band was attributed to the Si–O–Si symmetric stretching vibration mode of Q^3^ species [30]. In the meantime, the band also originated from the LO mode of nickel oxide [31]. An analogous situation occurred in the high-frequency region. The 1100 cm^−1^ band was assigned to Si–O stretching vibration modes of Q^3^ species [30], and the characteristic peak was also attributed to 2LO mode of NiO [31]. According to the surface morphology of pure Ni_3_Si alloy (Figure 4d), the discontinuous layer consisted of spherical particles, and the morphology was in accord with that of NiO [32]. This meant that NiO indeed existed on the worn surface after the friction test, but the oxidation of Ni_3_Si alloy was prone to form an external NiO layer and an internal SiO_2_ layer [1,21]. In other words, the observed worn surface was the newly oxidized layer, and the entire surface suffered serious oxidation wear. Therefore, the wear rate of pure Ni_3_Si alloy increased sharply, and was the highest at 600 °C. 

With the addition of Ti, the Raman spectra showed peaks located at 548 cm^−1^, and the band could be assigned to the Ti-O vibrations and the Si–O–Si bending vibrations [33,34]. In addition, a new band observed at 1016 cm^−1^ was attributed to the Si–O stretching of Q^2^ units [35]. This presented that the oxidation of the alloy with Ti addition was more severe than that of pure Ni_3_Si alloy. The oxide layer formed on the surface of the alloy with 5% Ti addition, and thus the alloy had the lowest friction coefficient and wear rate among the alloys at 600 °C. The alloy with the addition of 10% Ti had a similar composition as the alloy with 5% Ti addition. However, the intensities of the peaks were lower, and this could result from the delamination of the worn surface. The delamination led to a higher wear rate, but the medium wear rate was acquired by the presence of a large amount of oxide debris on the worn surface (Figure 6d). Meanwhile, the accumulated oxides acted as a lubricant in reducing the friction, and the alloy with 10% Ti addition exhibited medium friction coefficient at 600 °C.

**Figure 8:**
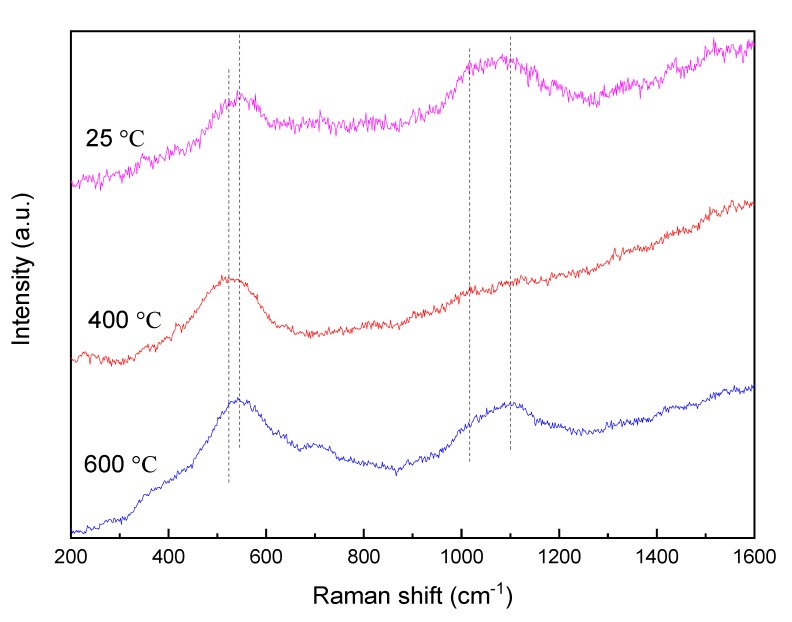
Raman spectra of the alloy with 5% Ti addition at different temperatures.

The alloy with 5% Ti addition had the lowest wear rate among the alloys at high temperatures, and the Raman spectra of the worn surfaces of the alloy at different temperatures are shown in Figure 8. The Raman spectra of the alloy at 200 °C was not given due to the similar curves between the temperature of 25 and 200 °C. It was clear that the main composition of the worn surface of the alloy was still silicon oxide at room temperature, which was similar to that at high temperature (600 °C). The obvious difference was that the peak intensity of the high-frequency region was relatively higher than that of the low-frequency region. It was well-known that the bands in the high-frequency region associated with the stretching vibration mode of nonbridging oxygens, and the bands in the low-frequency region associated with the vibration of bridging oxygens. This indicated that the oxidation occurred only at the surface, and there was little bonding between the oxidation units (silicon-oxygen tetrahedrons) on account of the low content of bridging oxygens. Therefore, the degree of the oxidation of the alloy was relatively low at room temperature. 

When the temperature was up to 400 °C, the band shifted to a lower wavenumber from 560 to 527 cm^−1^ in the low-frequency region. The decreasing shift of the band could be attributed to an increase in the average Si–O–Si angle that occurred as a result of silicate polymerization [29,36]. Moreover, the peak intensity of the low-frequency region was significantly higher than that of the high-frequency region. The good high-temperature strength of the alloy provided the desired wear resistance, and the alloy was hard to wear at 400 °C. The species of oxides were squeezed out and accumulated on the surface during the friction process. Further, the strong bonding between the oxides was achieved by the formation of bridging oxygens, and thus the relative intensity of the bands was much higher in the low-frequency region than that in the high-frequency region. As the temperature increased further, the peak intensities of the alloy were high both in the low-frequency and the high-frequency regions. The reason for this was that the severe oxidation was beneficial to the bonding between the silicon-oxygen tetrahedrons, and the re-oxidized regions resulted in varying degrees of oxidation of the surface. Finally, the alloy with 5% Ti addition exhibited the best tribological property at 600 °C as compared to the other alloys.

## 4. Conclusions

The effect of the addition of Ti on tribological behaviors of Ni_3_Si alloy has been investigated under high temperatures. The main conclusions are summarized as follows:

- The friction coefficient of Ni_3_Si alloys showed a declining trend with the increase of temperature. However, the reverse trend was observed between the alloys with Ti addition, and this was due to the different contents of Ti.
- The wear rates of the alloys had a little change in the temperature range of 25–400 °C, but the wear rates increased sharply at 600 °C. There were two reasons for this: First, the yield strength reached a maximum at 400 °C, and then decreased with the increase of temperature; second, the severe oxidation occurred at 600 °C. The Ni_3_Si alloy with 5% Ti addition showed the best wear resistance at high temperatures as compared to pure Ni_3_Si alloy and with 10% Ti addition. The wear rates of the tested alloys were in the magnitude of 10^−5^ mm^3^/m.
- The wear mechanism of pure Ni_3_Si alloy was abrasive wear at low temperature, and oxidation wear at high temperature. When the addition of Ti content increased, the wear mechanisms of the alloys changed from abrasive wear to fatigue wear at low temperature, and oxidation wear and fatigue wear at high temperature.
